# Effect of Bio MTA plus & ProRoot MTA pulp capping materials on the regenerative properties of human dental pulp stem cells

**DOI:** 10.1038/s41598-025-88816-5

**Published:** 2025-02-08

**Authors:** Khaled Mohamed Ayoub, Mohamed Mokhtar Nagy, Riham Mohamed Aly, Ghada Nour El Deen, Karim El-Batouty

**Affiliations:** 1 Endodontic Department, Faculty of Dentistry, Badya University, Cairo, Egypt; 2 Endodontic Department, Faculty of Dentistry, Ain Shams University, Cairo, Egypt; 3 Endodontic Department, Faculty of Dentistry, Galala University, Suez, Egypt; 4 Department of Basic Dental Science, Oral and Dental Research Institute, National Research Centre, Cairo, Dokki Egypt; 5 Stem Cell Laboratory, Center of Excellence for Advanced Sciences, National Research Centre, Cairo, Egypt; 6 Molecular Genetics and Enzymology Department, Human Genetic and Genome Research Institute, National Research Centre, Cairo, Dokki Egypt

**Keywords:** Cell biology, Mesenchymal stem cells

## Abstract

The aim of the present study was to investigate the effects of the biological properties of hDPSCs exposed to Bio MTA+ & ProRoot MTA pulp capping materials on the proliferation and odontogenic differentiation of hDPSCs. Human dental pulp stem cells (hDPSCs) were isolated from impacted third molars. Extracts of Bio MTA + and ProRoot MTA were prepared at a 1:1 ratio. The effects of the extracts on hDPSCs cytotoxicity and proliferation were assessed via a CCK-8 assay. Annexin V expression was investigated to assess the effects of both materials on the induction of apoptosis. The effects of ProRoot MTA and Bio MTA + extraction media on the stemness properties of hDPSCs were assessed via real-time quantitative PCR, and the expression of odontogenic markers (RUNX2, DMP1 & DSSP) was analyzed via RT‒PCR Alizarin Red staining. Cells exposed to Bio MTA + had the greatest degree of proliferation. The results of Annexin V staining indicated that Bio MTA + caused the least amount of apoptosis. RUNX2, DMP1 and DSSP were highly expressed by Bio MTA + and indicated successful odontogenic differentiation. Compared with ProRoot MTA, Bio MTA + exhibited an exceptional level of cytocompatibility, as well as advantageous bioactivities, including the preservation of stemness and an increase in the proliferation capacity of hDPSCs. In addition, it demonstrated favorable bioactive properties by stimulating odontogenic differentiation. Bio MTA + offers significant advantages in terms of biocompatibility, bioactivity, and regenerative potential, making it an excellent choice for procedures aimed at preserving or regenerating dental pulp tissue. However, additional research is required to address the lack of in vivo validation, as replicating physiological conditions is crucial for accurately assessing clinical outcomes and comparing them with results obtained from in vitro experiments.

## Introduction

In dentistry, pulp capping is a technique used to promote the establishment of reparative dentin and to preserve the vitality of the pulp by sealing the exposed vital pulp^1^. The ability of a material to induce the differentiation and maturation of dental pulp stem cells depends on its bioactivity and biocompatibility.

The efficacy of vital pulp therapy hinges upon a multitude of factors. The expansion and ability of stem cells to differentiate into both dental and bone tissues are crucial. Human dental pulp stem cells (hDPSCs) can be extracted from the dental pulp of primary, immature permanent, and mature permanent teeth in young adults. hDPSCs have garnered significant interest in the fields of tissue engineering and regenerative medicine because of their accessibility and ability to differentiate into multiple cell lineages. Human dental pulp stem cells (hDPSCs) can transform into several types of tissues, such as cartilage, bone, adipose, vascular, and nerve tissues. They have been intensively researched for their potential in treating conditions such as bone abnormalities, stress urine incontinence, retinal degenerative disorders, and other diseases.

Mineral trioxide aggregate (MTA) has become the preferred dental pulp capping material because of its sealing properties, biocompatibility, and antimicrobial effects^2^. It induces pulp healing and regeneration of dentin by promoting the release of cytokines and interleukins. However, the major drawbacks of MTA when used as a pulp capping material are its long setting time, high solubility, difficulty in manipulation, and discoloration of the tooth^3,4^. Another variant of MTA is ProRoot MTA (Dentsply, USA), which is presently a favored option for root repair^5^. In accordance with the manufacturer, ProRoot MTA is a hydraulic calcium-silicate cement derivative of type I ordinary Portland cement with a 4:1 ratio of bismuth oxide added for radiopacity.

Nevertheless, ProRoot MTA presents several undesirable characteristics, such as a long setting time, difficulty in manipulation and insertion, and high cost^6^. Owing to these drawbacks, a new calcium silicate cement, Bio MTA Plus (Bio MTA+) (CERKAMED, Poland), was recently introduced to the market, offering a similar composition as the original MTA. Compared with traditional MTA, Bio MTA + contains hydroxyapatite and nanoceramic particles in its composition; however, it offers advantages such as faster setting and easier manipulation^7,8^. In addition, Bio MTA + has recently emerged as a promising choice for pulp-capping procedures because of its exceptional biocompatibility and physicochemical qualities. It can shield nerves in the pulp from external stimuli and enhance the growth and development of hDPSCs into the odontogenic lineage^9^.

The enhanced role of Bio MTA + in odontogenic regeneration is closely tied to its superior bioactivity and ability to promote tissue healing and repair. As a calcium silicate-based material, it releases bioactive ions such as calcium and silicon, which play a key role in stimulating odontogenic differentiation of stem cells, such as human periodontal ligament stem cells (hPDLSCs). This action facilitates the regeneration of the dentin-pulp complex and supports mineralized tissue formation, such as dentinal bridges^10^. Bio MTA + bioactivity is demonstrated by its ability to form hydroxyapatite on its surface when in contact with biological fluids. This hydroxyapatite layer mimics natural mineralized tissues and enhances the sealing properties of the material. Compared to traditional materials like AH + Bioceramic, Bio MTA + provides superior cell adhesion, proliferation, and differentiation, making it ideal for promoting tissue regeneration in endodontic applications^10^. Additionally, Bio MTA + exhibits notable immunomodulatory properties by regulating inflammatory responses, reducing pro-inflammatory cytokines, and increasing anti-inflammatory cytokines. This property is crucial in creating an environment conducive to healing and regeneration, particularly in inflamed or compromised tissues^11^. Thus, the enhanced bioactivity and immunomodulatory potential of BioMTA + make it a powerful material for promoting odontogenic regeneration, ensuring better clinical outcomes in regenerative endodontics^12^.

Hence, it is logical to hypothesize that Bio MTA + could augment the proliferation and differentiation of hDPSCs, consequently enhancing the efficacy of vital pulp therapy. However, to our knowledge, few studies have investigated the specific effects of Bio MTA + on the regenerative behavior of hDPSCs. Thus, the objective of the current study was to compare the viability, proliferation, stemness, and differentiation responses of human dental pulp stem cells to two calcium silicate-based materials, Bio MTA + and the widely used ProRoot MTA.

## Materials & methods

### Material preparation

The preparation methods and ratios for ProRoot MTA (Dentsply International Inc., DE, USA) and Bio MTA+ (Cerkamed Medical Company, Poland) were specifically designed to enhance their bioactivity, biocompatibility, and regenerative potential, ensuring effective odontogenic differentiation of DPSCs. Both materials were prepared following the manufacturer’s instructions. Additionally, the extraction medium ratios for both materials were determined based on the study by Manaspon et al.,^13^ and Esen et al.,^14^ which examined the effects of similar calcium silicate-based materials on dental pulp stem cells. Bio MTA + was formulated with improved properties, including faster hydration, smoother handling, and superior ion release compared to ProRoot MTA. To prepare Bio MTA+, the entire contents of the powder vial were mixed with 1 to 2 drops of the liquid on a mixing plate for 30 s, achieving a soft plasticine (modeling putty) consistency. In contrast, ProRoot MTA, known for its more granular texture, required careful preparation to achieve proper consistency and bioactivity. For this, a 3:1 powder-to-saline ratio was used, resulting in a putty-like consistency. The mixtures were then poured into cylindrical molds measuring 2.5 mm in height, 5 mm in diameter, and 9 mm in length. The acquired discs were subjected to 24 h of exposure to ultraviolet (UV) radiation within a safety cabinet. The discs were inserted into 50-ml conical tubes and subjected to extraction via either growth medium (DMEM) or odontogenic induction medium. This process took place in an incubator for 24 h at 37 °C and 5% CO2. The procedure followed the guidelines outlined in ISO 10993-12:2007 292. The extraction medium from each sample was filtered twice via a 0.2 μm filter to eliminate any remaining tiny particles.

### Sample collection and dental pulp stem cell isolation

The experimental protocol of this research was reviewed by the Ethical Committee of the Faculty of Dentistry Ain Shams University and was granted approval (FDASU-Rec ID 052329). Healthy human premolars (*n* = 4) were obtained from healthy individuals aged 14 to 18 years who were undergoing extraction for orthodontic purposes. After providing informed consent, the human dental pulp stem cell (hDPSCs) isolation protocol followed that of Gronthos et al.^15^ with some modifications^16,17^. The extracted premolars were transferred to Dulbecco’s modified Eagle’s medium (DMEM) containing antibiotics at a concentration of 100 U/ml penicillin and 100 mg/ml streptomycin. The premolars were split open, and the pulp tissue was delicately removed and washed with phosphate-buffered saline (PBS) containing penicillin and streptomycin. The tooth pulp tissue was sliced into small fragments and then exposed to 2 mg/ml collagenase type I (Serva Electrophorese, Germany) for 15 min at 37 °C while being continuously agitated through shaking. The single-cell suspensions were subjected to culture in a humidified environment of 5% CO2 at 37 °C in DMEM (Lonza), 15% fetal bovine serum (Lonza), 10,000 U/ml penicillin and 10,000 ng/ml streptomycin. The cellular specimens were routinely examined via an inverted microscope, and the culture media was replenished periodically. The cells were passaged when they reached 70% confluence via TrypleSelect^®^. The cells obtained from the third and fourth passages were used in the subsequent experiments. Once the cells reached 70% confluency, the cell culture media was replaced with extracted culture media diluted to a 1:1 ratio^18,19^.

### Characterization of the isolated cells

#### Flow cytometric analysis (FACS)

Cytomics FC500 flow cytometer (Beckman Coulter, USA) was used to conduct FACS analysis for CD90, CD105, and CD34 to verify the existence of mesenchymal stem cells (MSCs) in the isolated cells. The expression of MSCs markers was quantified using CXP software version 2.2 according to the following procedures (Beckman Coulter, USA). A total of 1106 cells per milliliter were subjected to incubation at 4 °C in the absence of light with 10 milliliters of monoclonal antibodies targeting CD34 to eliminate the possibility of hematopoietic origin. CD90 and CD105 were also used to verify the identity of mesenchymal stem cells (Beckman Coulter, USA) (Hanna et al. 2023, 2024). An isotype group was used as the control group. After 20 min of incubation, tubes containing the cells treated with monoclonal antibodies were filled with 2 ml of PBS containing 2% fetal bovine serum (FBS). Upon centrifugation at 2500 rpm for 5 min, the supernatant was removed, and the cells were reconstituted in 500 ml of PBS containing 2% FBS.

### Assessment of multilineage differentiation potential

A lineage-specific staining technique was used to assess the multilineage differentiation capability of hDPSCs. Following resuspension in culture medium, 5,000 cells were plated in 12-well culture plates. Upon adherence to the plates, the medium was replaced with osteogenic, adipogenic, or chondrogenic differentiation medium. The cells were then grown for 3 weeks to evaluate their ability to differentiate effectively. A solution of 0.3% Oil Red O was used to stain hDPSCs to identify the existence of extracellular lipid droplets. Chondrogenic differentiation was identified by the detection of sulfated glucose amino glycans by Alcian blue staining. To evaluate the ability of hDPSCs to generate osteogenic lineages, osteoblast-like cells were stained with a 1% Alizarin Red S solution (Sigma‒Aldrich).

### Assessment of cell viability and proliferation via the CCK-8 assay

To evaluate the effects of the extraction media of both dental pulp capping materials and the control group on hDPSCs proliferation, a cell counting kit-8 (WST-8/CCK-8) was used. The number of cells used for proliferation testing was 5000 following the manufacturer’s instructions^20^. Proliferation testing was performed on days 1, 3 and 7.

### Annexin V assay

Apoptosis Kit with Annexin V-FITC and propidium iodide (PI) for flow cytometry was conducted to separate apoptotic cells (early) and dead cells (late apoptotic cells). The monoclonal antibodies detect phosphatidylserine externalization, which is an apoptosis marker, in both apoptotic and dead cells via recombinant Annexin V, which is conjugated to green, fluorescent FITC dye, and propidium iodide (PI), which is conjugated to red fluorescence. Human dental pulp stem cells were reconstituted in 20 µl of binding buffer and thereafter exposed to 5 µl of PI and 10 µl of Annexin V-FITC for 20 min in a dry chamber set at 25 degrees Celsius. Upon exposure to both probes, apoptotic cells presented green fluorescence, whereas dead cells presented both red and green fluorescence, and living cells presented minimal or no fluorescence. Analysis of the flow cytometry data was conducted via the Navios software developed by Beckman Coulter.

### Cell cycle analysis

Flow cytometry cell cycle assay was used to monitor the phases of the cell cycle. The assay was useful for determining the percentage of examined cells in each stage of the cell cycle (G1– S – G2 & M phases). Human dental pulp stem cells were seeded at a density of approximately 1.19 × 10^3^ cells/cm^2^ and subsequently harvested after 2 days. A solution of 70% ethanol was subsequently added to the cells for 12 h. Following washing with PBS, a total of 2 × 10^5^ cells were stained with 200 µl of propidium iodide for 30 min at 37 °C in the dark. The samples were analyzed via a Cytomics FC500 flow cytometer (Beckman Coulter, USA) and compared with control cells that were cultured in DMEM only.

### Assessment of the effect of ProRoot MTA and Bio MTA + extraction media on stemness properties via real-time quantitative PCR

The stemness of hDPSCs was assessed by analyzing the expression of stemness markers (*OCT4* & *SOX2*) after culture in ProRoot MTA and Bio MTA + extraction media. The differential expression of both stemness markers was analyzed via real-time quantitative polymerase chain reaction. The primers used for this study were ***Oct4*** 5’-ATCAAAGCTCTGCAGAAAGAACT-3’ (forward), 5’-GCTTACACATGTTCTTGAAGCTAA-3’ (reverse), ***SOX2***: 5’-ATAATAACAATCATCATCGGCGG-3’ (forward) and 5’-AAAAGAGAGAGGCAAACTG-3’ (reverse). The data were analyzed via the relative expression software tool (REST) with the automatic cycle threshold (Ct) option. The CT method was employed to calculate the relative expression (RE) of the sample genes, utilizing GAPDH as an internal control. Three replicate quantitative reverse transcription polymerase chain reaction (qRT‒PCR) experiments were conducted. The data are presented as the average values and standard errors of the means.

### Induction of odontogenic differentiation

Odontogenic differentiation was initiated by replacing the culture media with odontogenic induction media containing DMEM, 10% fetal bovine serum, 5 mM β-glycerophosphate, 100 µM L-ascorbic acid 2-phosphate, 0.01 µM dexamethasone, and 2 mM L-glutamine for 14 days. hDPSCs were seeded at a density of 1 × 10^4^ cells/well in 12-well culture plates. After 1 day, when hDPSCs had reached approximately 30% confluency, they were cultured in ProRoot MTA and Bio MTA + extraction odontogenic induction media diluted to a concentration of 1:1^18,19^. For the negative and positive controls, hDPSCs were cultured in DMEM alone or in odontogenic induction medium, respectively.

### Assessment of the odontogenic differentiation of hDPSCs cultured in ProRoot MTA and Bio MTA + extraction media

#### Alizarin red staining

Alizarin red staining was employed to verify the successful differentiation of hDPSCs, as evidenced by in vitro mineralization at 7 and 14 days. The cells were fixed for 40 min with 4% paraformaldehyde (SigmaAldrich, USA) and rinsed twice. Then, 1% Alizarin Red S (Sigma‒Aldrich, USA) was added for 20 min. The mineralized deposits were observed via inverted light microscope (Leica, Germany).

#### Real-time polymerase chain reaction (PCR) analysis of odontogenesis-related gene expression

The relative gene expression between the different groups and the control was determined to better evaluate the effects of both pulp capping materials on the odontogenic differentiation of hDPSCs. Total RNA was isolated via a Directzol RNA Miniprep Kit (Zymo Research), and cDNA synthesis was performed via a RevertAid First Strand cDNA Synthesis Kit (Thermo Scientific) according to the manufacturer’s instructions. After odontogenic induction for 7 and 14 days, the cell phenotype markers employed in this study were runt-related transcription factor 2 (*RUNX2*), direct stock purchase plan (*DSPP*) and dentin matrix acidic phosphoprotein 1 (*DMP1*). Samples were normalized to total RNA content and to the housekeeping gene GAPDH, and all experiments were performed in triplicate. Fold differences in gene expression were calculated via the delta‒delta Ct method (2ΔΔCt) [16]. The sequences of primers used were as follows: *RUNX*2: 5` -CACTGGCGCTGCAACAAGA-3` (forward) and 5` -CATTCCGGAGCTCAGCAGAATAA-3` (reverse); *DMP1*: 5` -AGGAAGTCTCGCATCT CAGAG-3` (forward) and 5` -TGGAGTTGCTGTTTTCTGTAGAG-3` (reverse); *DSPP*: 5` -TCACAAGGGAGAAGGGAATG-3` (forward) and 5` -TGCCATTTGCTGTGATGTTT3` (reverse); and GAPDH (the internal control gene) 5’-CATGAGAAGTATGACAACAGCCT-3’ (forward) and 5’-AGTCCTTCCACGATACCAAAGT-3’ (reverse).

### Statistical analysis

All the data were analyzed via Statistical Product and Service Solutions (SPSS) 20.0 software (IBM, Armonk, NY, USA). Data were presented, and a suitable analysis was performed according to the type of data obtained for each parameter. For the analysis of proliferation, the normality of the distribution parameters was assessed using the one-sample Kolmogorov‒Smirnov test. For datasets with a normal distribution, one-way ANOVA was performed, followed by post hoc tests to evaluate differences between groups. For RT‒PCR analysis, data were analyzed using one-way ANOVA with Bonferroni post hoc correction for multiple comparisons. P values are reported in the figures, with *P* < 0.05 considered statistically significant. Variability among replicates was addressed by including three replicates per group. Data were presented as mean ± standard deviation (SD) to accurately represent variability to ensure reproducibility and robustness in the reported outcomes. The following measures were implemented to ensure the reliability and reproducibility of the findings, accounting for potential variability and data integrity throughout the analyses. Missing data (for example, wells in the proliferation assay that did not yield a reading due to a pipetting error, plate reader malfunction, or contamination, etc…) were handled systematically. Cases with missing values were imputed using the mean value of available replicates. The proportion of missing data was negligible and did not impact the overall results.

## Results

### Successful isolation of hDPSCs

**Fig. 1:**
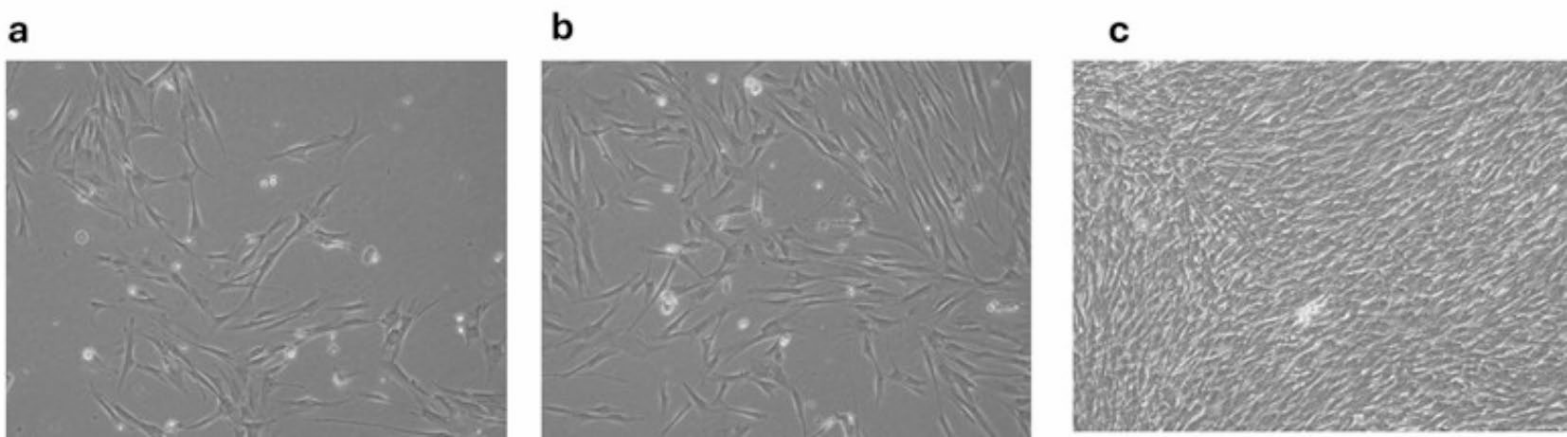
Morphological appearance of isolated hDPSCs. Scattered spindle-shaped cells started to appear nearly 5 days after isolation (**a**), and the number of cells started to increase (**b**) until they reached 80% confluency (**c**).

**Fig. 2:**
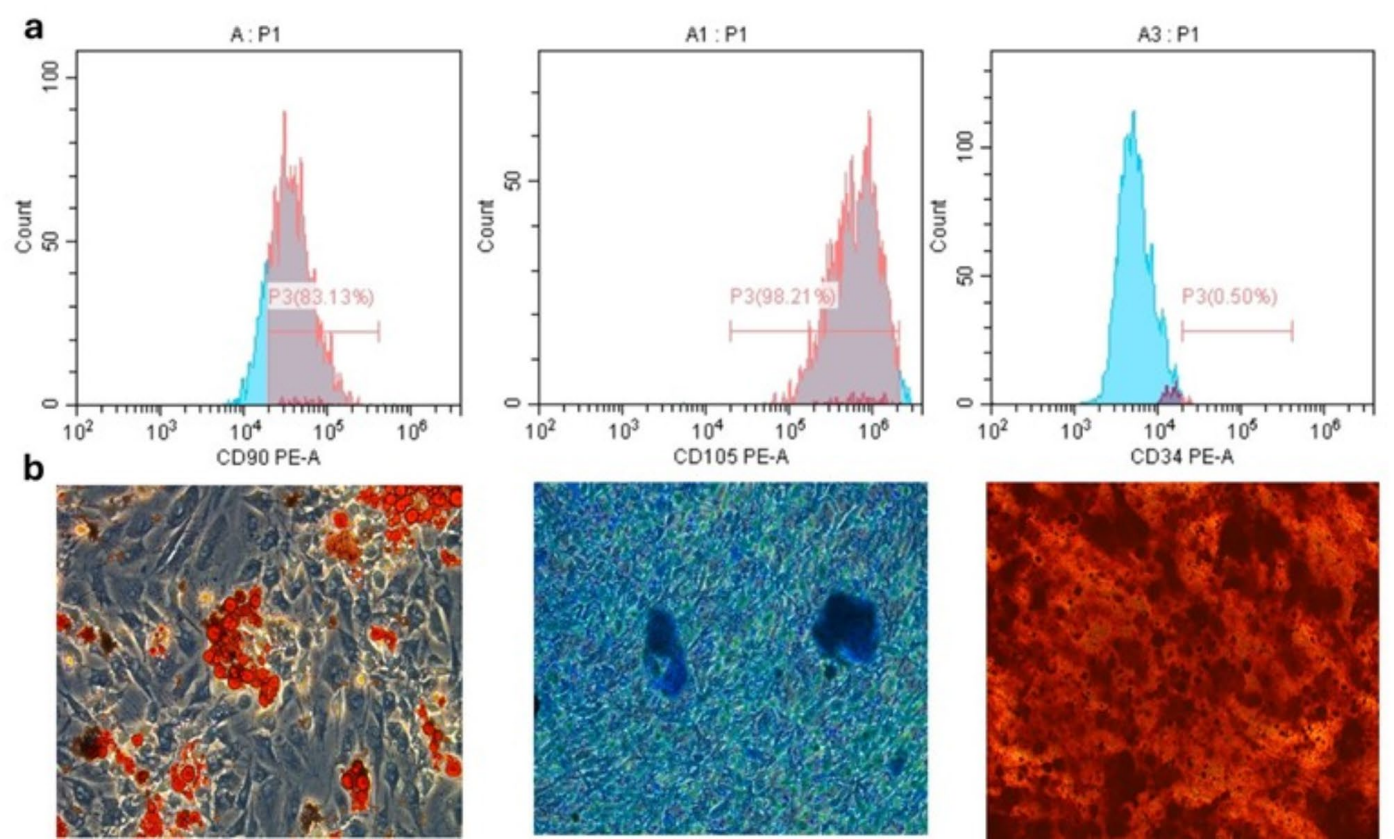
Characterization of isolated hDPSCs. (**a**) FACS analysis revealed that the isolated cells were positive for MSCs markers CD 90 and CD105 and negative for the hematopoietic stem cell marker CD34. (**b**) Multilineage differentiation potential of isolated hDPSCs. Isolated cells demonstrated positive staining for adipogenic (left), chondrogenic (middle) and osteogenic (right) lineages, confirming their mesenchymal stem cell identity.

The isolated cells exhibited the characteristic morphological appearance of stem cells. After isolation, the cells started to attach to the culture plates, demonstrating a spindle fibroblast-like appearance. The cells increased in number and reached 80% confluence nearly 5 days after isolation (Fig. 1). The stem cell identity of the isolated cells was confirmed via flow cytometric analysis of MSC markers, and the isolated cells were positive for CD90 (83.13%) and CD105 (98.21%) and negative for the hematopoietic marker CD34 (0.50%) (Fig. 2a). Moreover, the results were confirmed by successful differentiation into osteogenic, adipogenic and chondrogenic lineages (Fig. 2b).

### Effect of ProRoot MTA and Bio MTA + extraction media on the proliferation and viability of hDPSCs

**Fig. 3:**
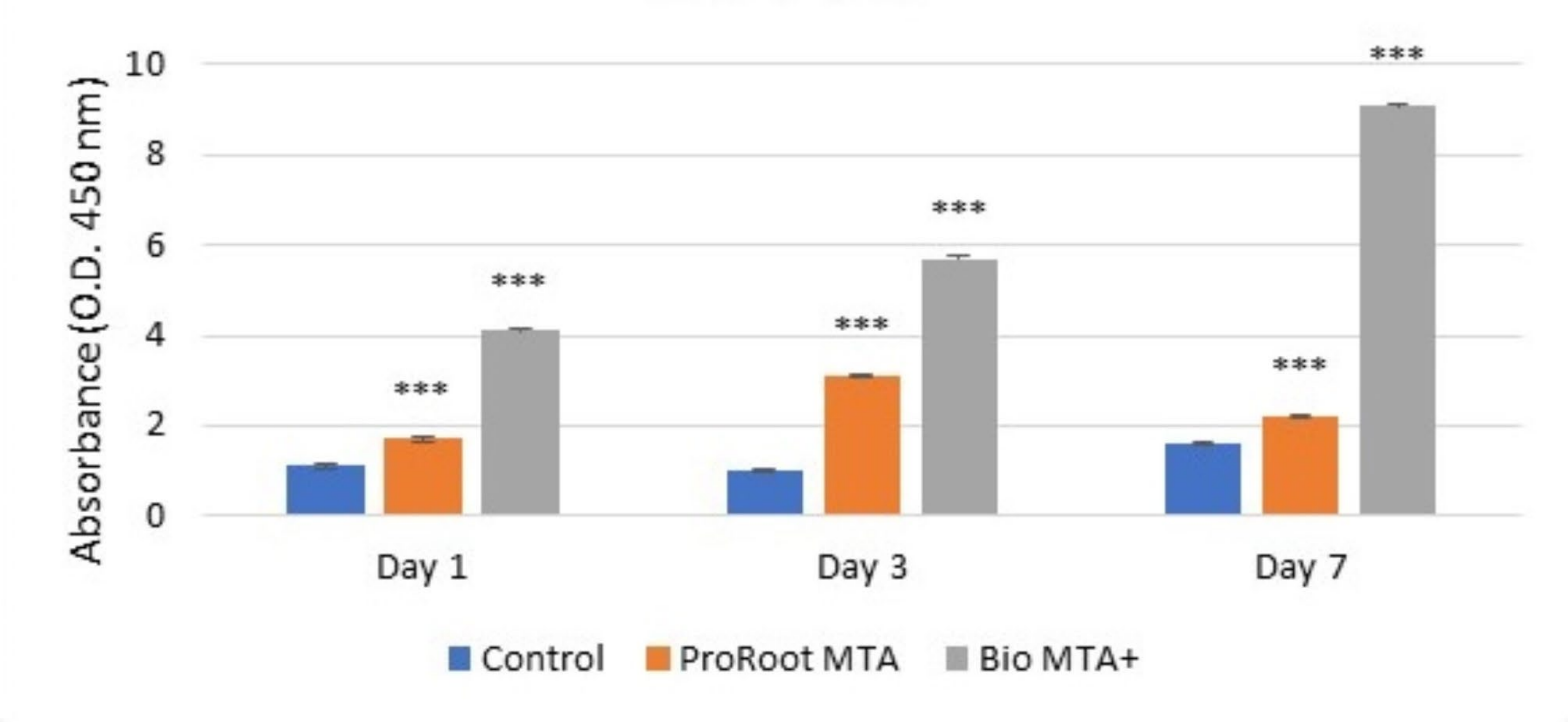
Bar chart representing the mean cellular proliferation of hDPSCs cultured with either ProRoot MTA or Bio MTA + extraction media in comparison with the control. ***p < 0.001 represents a significant difference compared with the control.

**Fig. 4:**
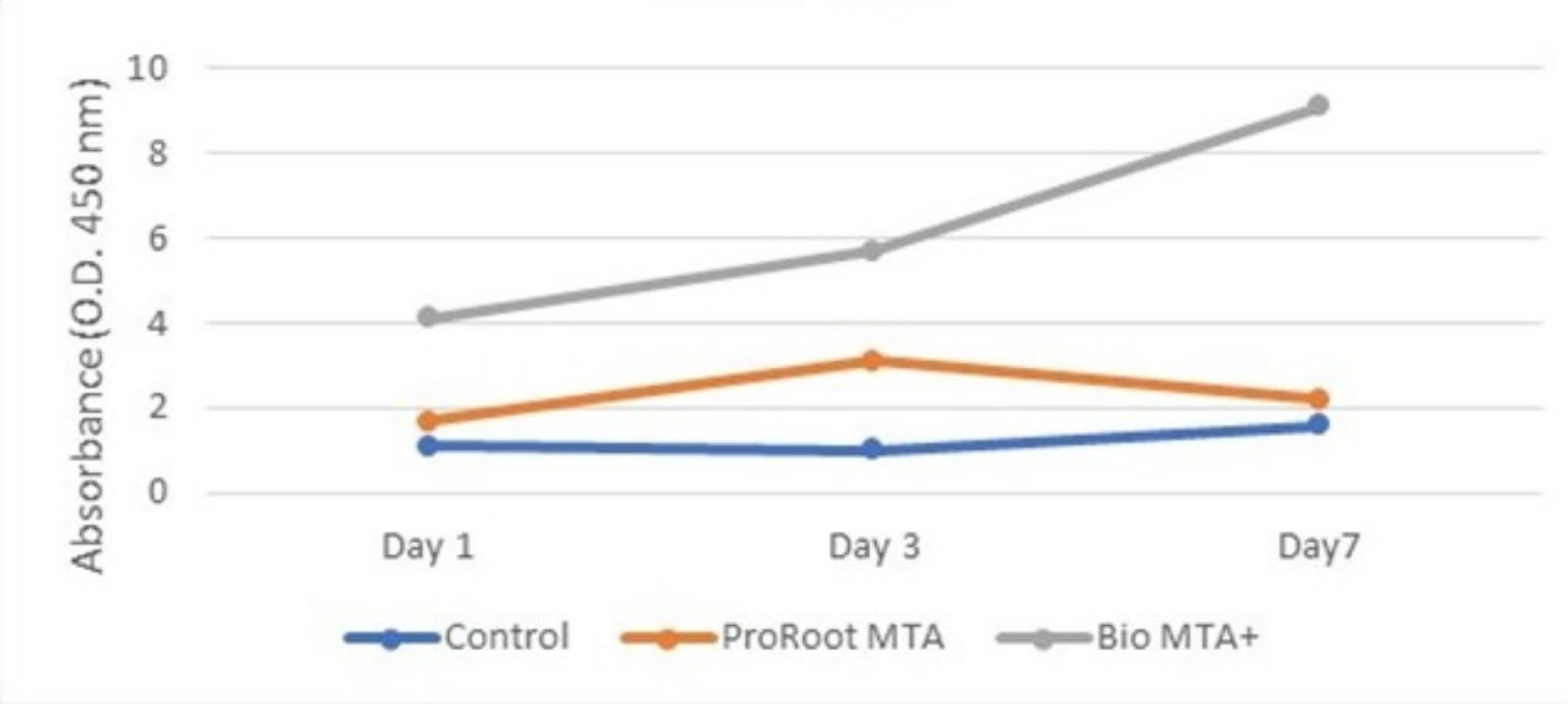
Cell proliferation varied with time and among the different materials. The cells exposed to Bio MTA + had the highest proliferation rate from days 1 to 7 (*P* < 0.001), whereas the cells in the control group had the lowest proliferation rate (*P* < 0.001) from days 1 to 7. The proliferation of cells exposed to ProRoot MTA was comparable to that of the control group on days 1 and 7 but was significantly greater on day 3 (*P* < 0.001).

The mean number of cells cultured in the Bio MTA + extraction media was the highest at days one, three and seven. These differences were statistically highly significant. There were statistically highly significant differences between {Bio MTA + vs. Control & ProRoot MTA+} at days one, three and seven. However, there were no statistically significant differences between the {Control group and the ProRoot} group at days one, three and seven. The mean differences in hDPSCs cultured in Bio MTA + extraction media were greater than those in hDPSCs control and ProRoot MTA extraction media on day one, day three and day seven by 17.5, 18.5, 17.1, 17.0, and 13.5 & 13.1, respectively, and vice versa (Table 1**&** Fig. 3). Moreover, the mean number of cells on day seven for the control group and hDPSCs cultured in ProRoot MTA extraction medium was the highest. These differences were statistically highly significant. There were statistically significant differences between day three and day one and between day seven and day one and day three for both the control and ProRoot extraction media. The mean differences in the number of hDPSCs on day three were greater than those on day one in the control and ProRoot MTA extraction media by 2.1 and 3.2, respectively, and vice versa. Additionally, the mean differences on day seven were greater than those on day one and day three in the control and ProRoot MTA groups by (8.2, 6.1) and (9.7 & 6.5), respectively, and vice versa. There were no statistically significant differences between days one, three and seven for hDPSCs cultured in the Bio MTA + extraction medium (Table 2**&** Fig. 4).

**Table 1:** Comparison of different pulp capping materials with respect to time-dependent increases in cell proliferation.

	group	N	Mean	SD	Median	Range		F	P Value	Sig.
						Min.	Max.			
Day one	Control^a, b^	6	77.1	1.1	76.9	75.8	78.8	94.9	<0.001	HS
	***ProRoot MTA*** ^***b, a***^	**6**	**76.0**	**1.7**	**75.4**	**74.9**	**79.3**			
	***Bio MTA+*** ^***c***^	**6**	**94.5**	**4.1**	**95.0**	**89.6**	**98.8**			
**Day three**	***Control*** ^***a, b***^	**6**	**79.2**	**1.0**	**79.5**	**77.4**	**80.0**	**40.8**	**<0.001**	**HS**
	***ProRoot MTA*** ^***b, a***^	**6**	**79.2**	**3.1**	**78.2**	**77.1**	**85.3**			
	***Bio MTA+*** ^***c***^	**6**	**96.2**	**5.7**	**98.3**	**85.5**	**100.8**			
**Day seven**	***Control*** ^***a, b***^	**6**	**85.3**	**1.6**	**85.4**	**82.8**	**86.9**	**11.9**	**0.001**	**HS**
	***ProRoot MTA*** ^***b, a***^	**6**	**85.7**	**2.2**	**85.7**	**83.4**	**88.0**			
	***Bio MTA+*** ^***c***^	**6**	**98.8**	**9.1**	**101.4**	**81.1**	**105.4**			

**Table 2:** Comparison of the time-dependent increase in cell proliferation among the material groups.

	Group	N	Mean	SD	Median	Range		F	P Value	Sig.
						Min.	Max.			
Control	Day one^a^	6	77.1	1.1	76.9	75.8	78.8	69.4	<0.001	HS
	***Day three*** ^***b***^	**6**	**79.2**	**1.0**	**79.5**	**77.4**	**80.0**			
	***Day seven*** ^***c***^	**6**	**85.3**	**1.6**	**85.4**	**82.8**	**86.9**			
**ProRoot MTA**	***Day one*** ^***a***^	**6**	**76.0**	**1.7**	**75.4**	**74.9**	**79.3**	**25.6**	**<0.001**	**HS**
	***Day three*** ^***b***^	**6**	**79.2**	**3.1**	**78.2**	**77.1**	**85.3**			
	***Day seven*** ^***c***^	**6**	**85.7**	**2.2**	**85.7**	**83.4**	**88.0**			
**Bio MTA+**	***Day one***	**6**	**94.5**	**4.1**	**95.0**	**89.6**	**98.8**	**0.65**	**0.538**	**NS**
	***Day three***	**6**	**96.2**	**5.7**	**98.3**	**85.5**	**100.8**			
	***Day seven***	**6**	**98.8**	**9.1**	**101.4**	**81.1**	**105.4**			

**Fig. 5:**
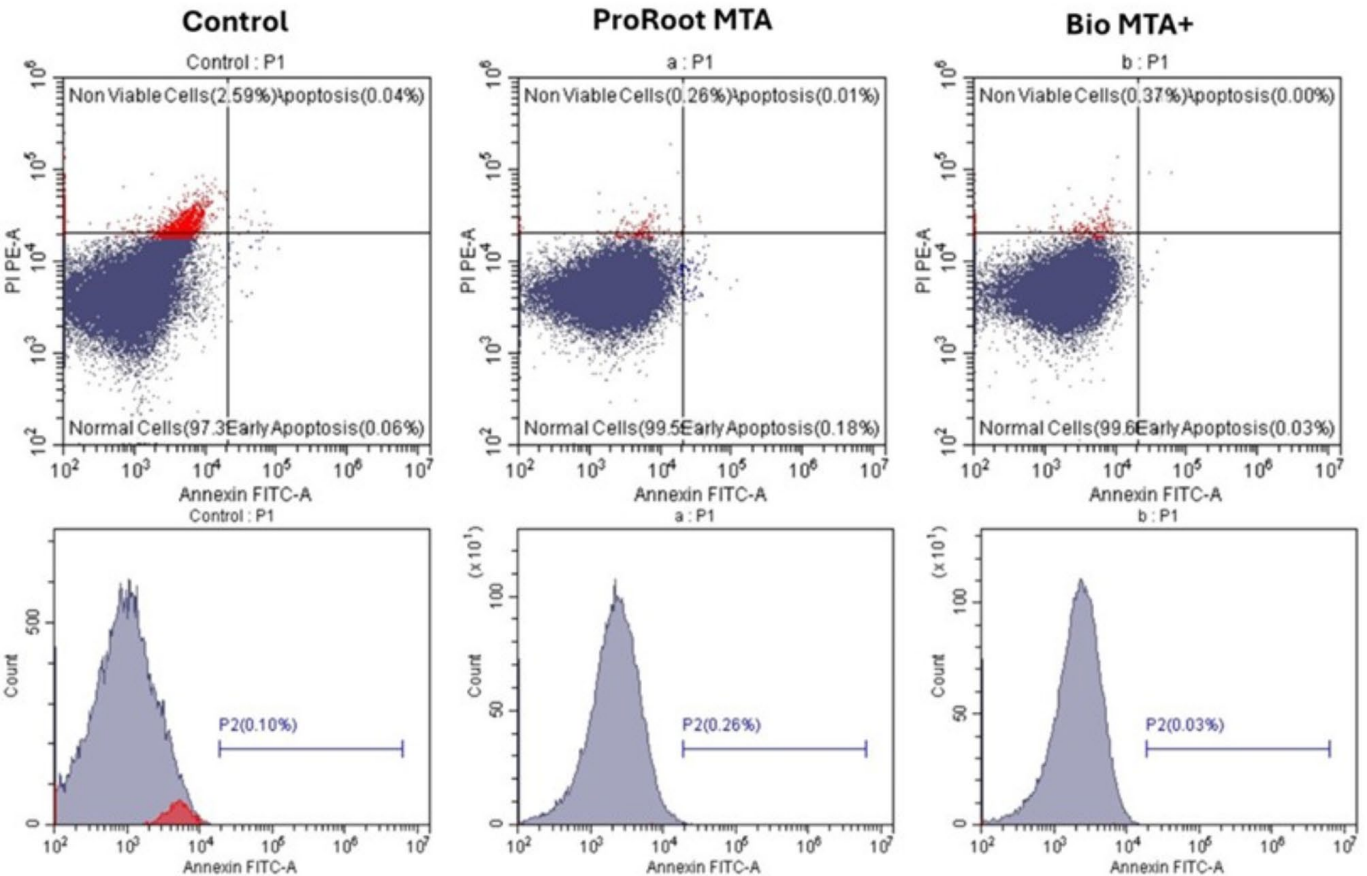
Flow cytometry analysis of Annexin V expression in hDPSCs cultured in ProRoot MTA (middle) and Bio MTA+ (right) extraction media in relation to the control (left).

### Annexin V analysis of hDPSCs cultured in ProRoot MTA and Bio MTA + extraction media

Annexin V analysis was used to evaluate the effect of ProRoot MTA and Bio MTA + extraction media on the proliferative activity of hDPSCs. The results revealed that hDPSCs cultured in DMEM supplemented with Bio MTA + extracts expressed the lowest percentage of cells in the late apoptotic stage (0.00%), nonviable cells (0.37%) and early apoptotic cells (0.03%). In contrast, ProRoot MTA revealed that the percentage of late apoptotic cells was 0.01%, that of nonviable cells was 0.26%, and that of early apoptotic cells was 0.18%. These results were compared with those of the control group, which revealed that the percentage of late apoptotic cells was 0.04%, the percentage of nonviable cells was 2.59%, and the percentage of early apoptotic cells was 0.06% (Fig. 5).

### Assessment of the cell cycle of hDPSCs cultured in ProRoot MTA and Bio MTA + extraction media

**Fig. 6:**
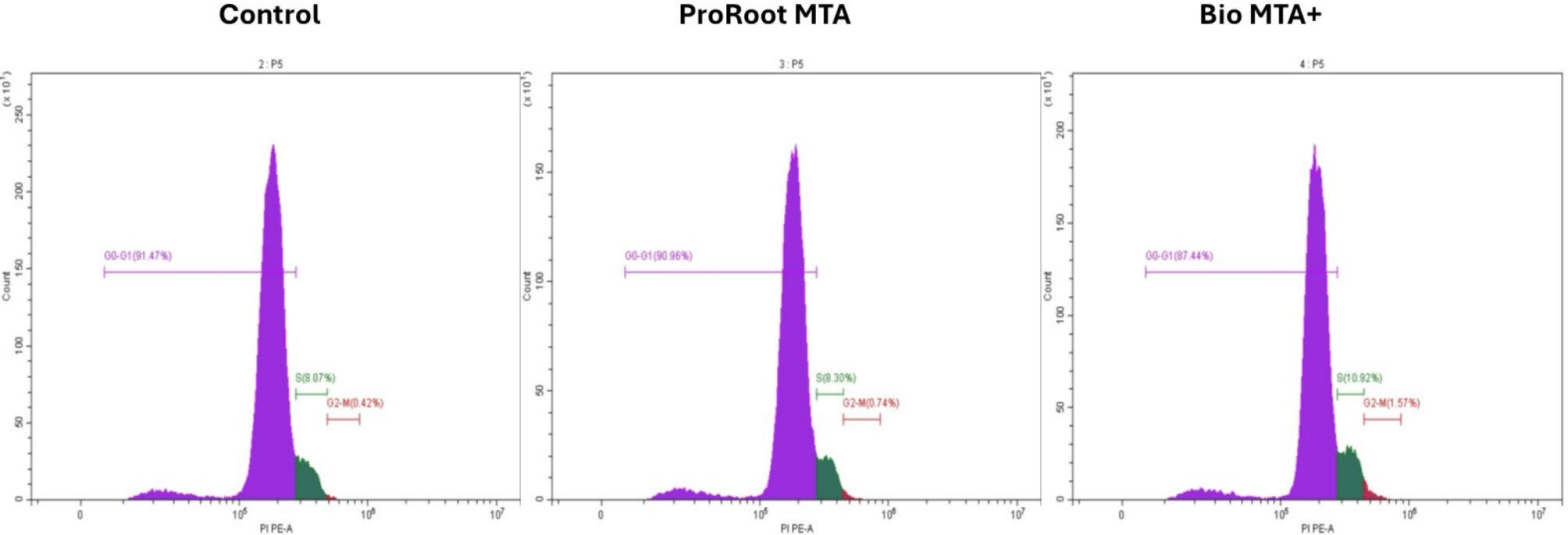
Cell cycle analysis of hDPSCs cultured in ProRoot MTA extraction medium (middle) and Bio MTA+ (right) extraction medium in relation to the control (left) by flowcytometry.

A cell cycle assessment was used to evaluate the effects of ProRoot MTA and Bio MTA + extraction media on the proliferative activity of hDPSCs. A cell cycle analysis of the Bio MTA + extracts revealed that the percentages of cells in G0 were as follows: G1 (87.44%), S (10.92%) and G2–M (1.57%). In contrast, the percentages of hDPSCs cultured in ProRoot MTA extraction media were G0–G1 (90.96%), S (8.30%) and G2–M (0.74%). The results were compared with those of the control group, which revealed that the percentages of cells in G0 were as follows: G1 (91.47%), S (8.07%) and G2–M (0.42%) (Fig. 6).

### Effect of ProRoot MTA and Bio MTA + pulp capping materials on the stemness of hDPSCs

**Fig. 7:**
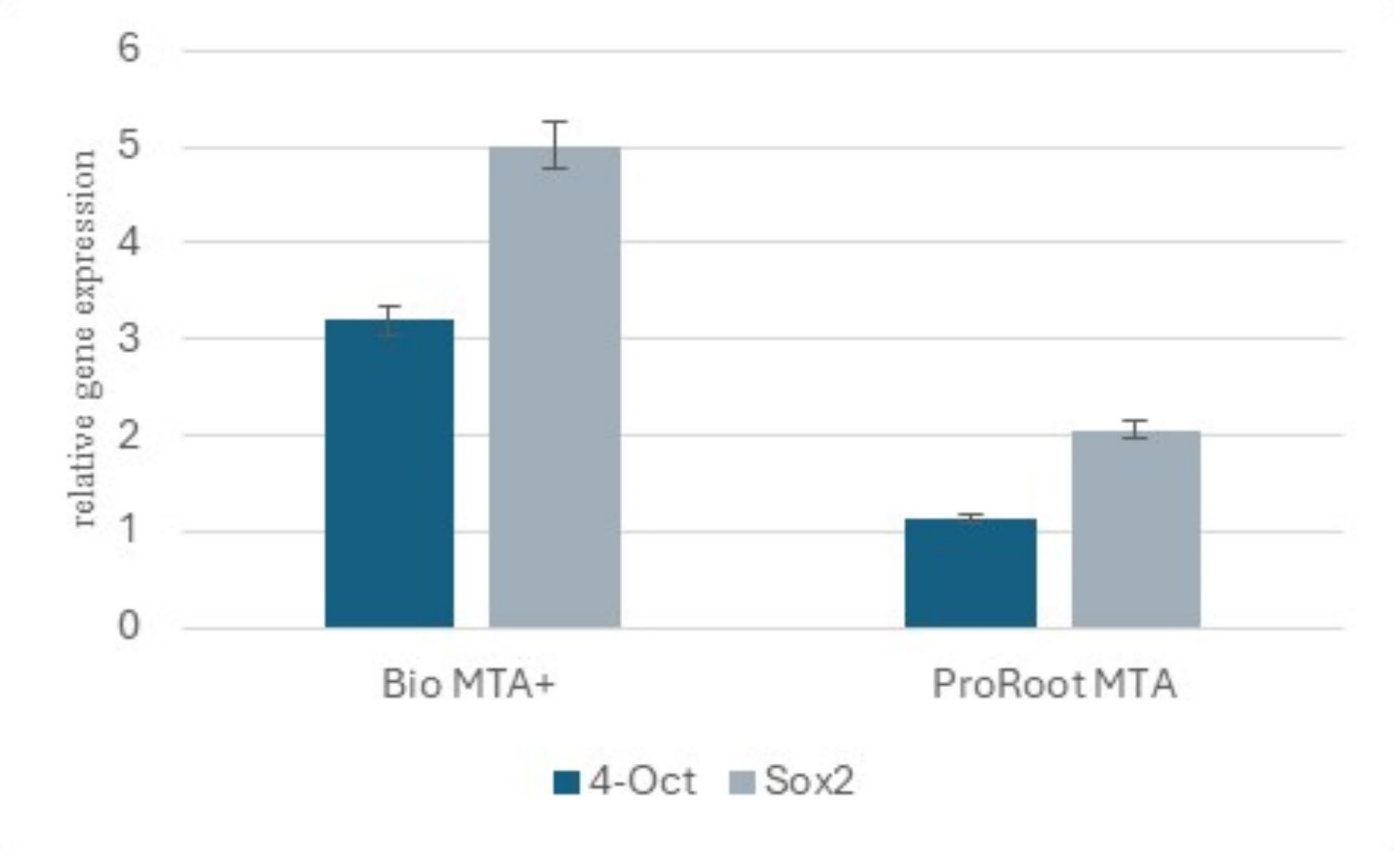
Expression of stemness genes in cells cultured in DMEM supplemented with either Bio MTA + or ProRoot MTA.

Quantitative real-time PCR analysis was implemented to identify the expression of stemness genes (Oct4 and *SOX*2). Both genes were expressed at higher levels in Bio MTA+, with relative fold changes of 3.19 ± 1.58 and 5.01 ± 2.59, respectively, than in ProRoot MTA, with relative fold changes of 1.13 ± 1.23 and 2.02 ± 0.94, respectively, in relation to those in dental pulp cells cultured in basal media (Fig. 7).

### Assessment of the effects of ProRoot MTA and Bio MTA + extraction media on the odontogenic differentiation potential of hDPSCs

#### Alizarin red staining

**Fig. 8:**
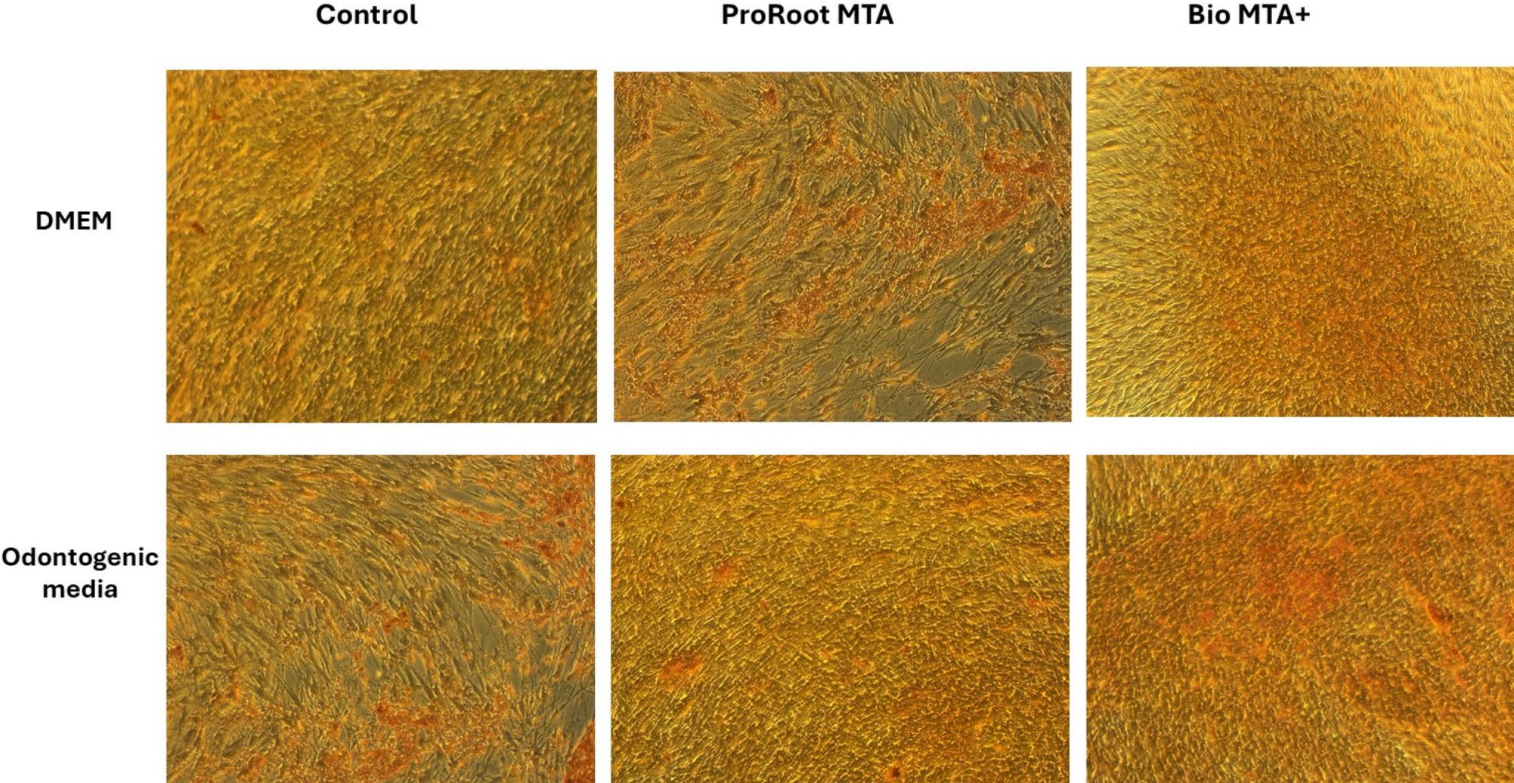
Alizarin Red staining of hDPSCs after induction of odontogenic differentiation via ProRoot MTA or Bio MTA + in either DMEM or odontogenic extraction media. (Magnification 10X). A comparison of the results of the alizarin red stain between ProRoot MTA and Bio MTA + in odontogenic medium revealed that the intensity of the alizarin red stain was greater with Bio MTA+. However, ProRoot MTA presented an apparent increase in intensity in DMEM compared with that of Bio MTA+.

After hDPSCs were cultured for 14 days in DMEM supplemented with ProRoot MTA extraction medium, staining with alizarin red revealed a mild positive reaction with only a few stained cells. However, when the cells were cultured in odontogenic medium supplemented with ProRoot MTA, the positive alizarin red stain was more intense. With respect to hDPSCs cultured in DMEM supplemented with Bio MTA+, a slight positive reaction was observed, with a few stained cells. However, when the cells were cultured in odontogenic media supplemented with Bio MTA+, the intensity of the positive Alizarin Red stain was apparently greater. Upon comparing the outcomes of alizarin red staining between ProRoot MTA and Bio MTA + in odontogenic media, it was evident that the intensity of the alizarin red stain was greater with Bio MTA+. The results were compared to those of both the negative and positive control groups. The negative control group demonstrated no staining, indicating a lack of mineralized nodules and therefore no differentiation. In contrast, the positive control group, which consisted of hDPSCs cultured in odontogenic media without either of the dental pulp capping materials, demonstrated positive staining with alizarin red, along with the presence of a few mineralized nodules (Fig. 8).

#### RT‒PCR

**Fig. 9:**
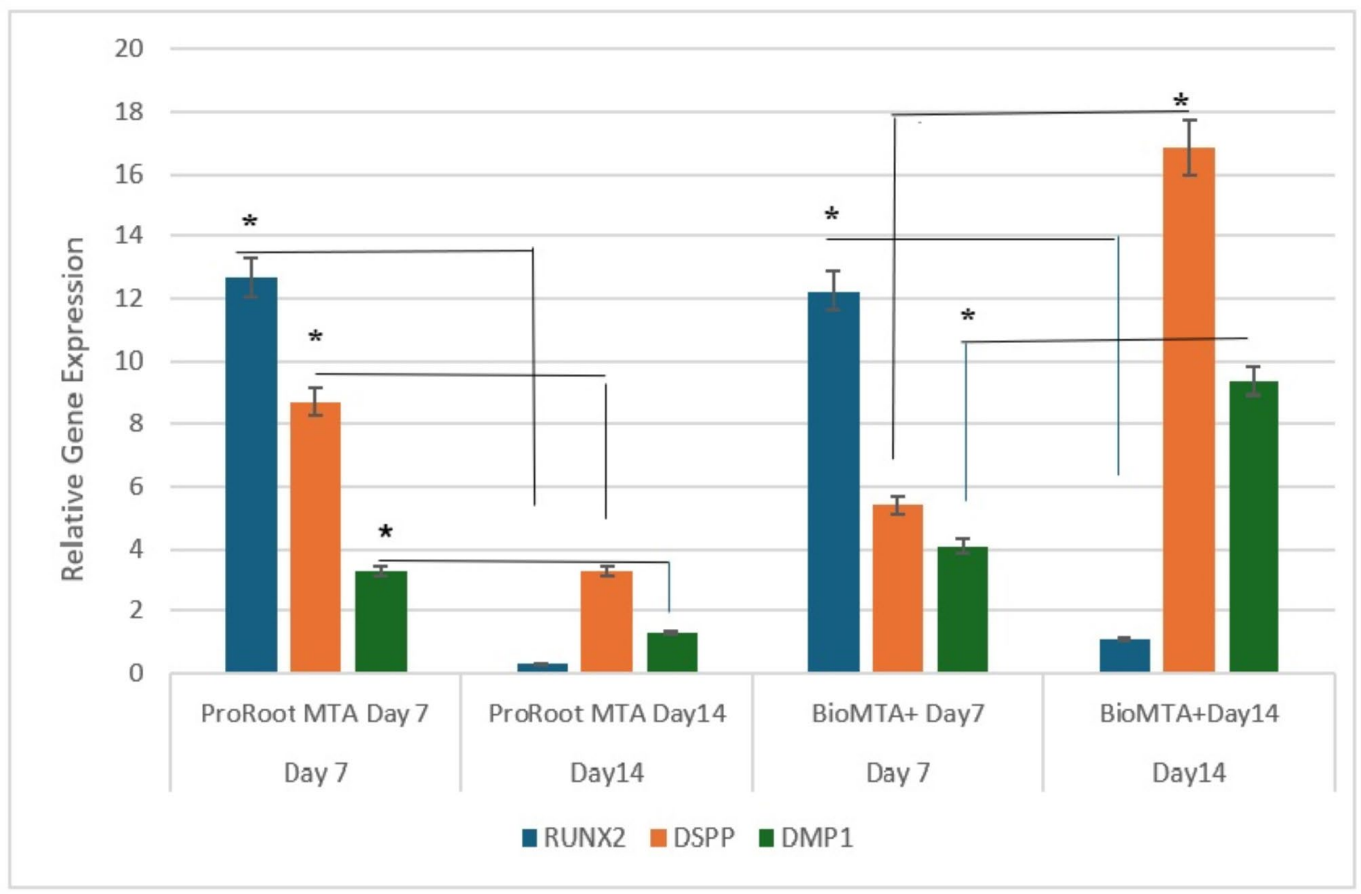
Expression of genes characteristic of the odontogenic differentiation of RUNX2, DSSP and DMP1 in cells cultured in odontogenic media supplemented with either ProRoot MTA or Bio MTA + on days 7 & 14. The results are presented as the mean ± SEM of three independent experiments (*n* = 3). Significance was calculated in relation to hDPSCs cultured in odontogenic media; *P < 0.05.

The odontogenic differentiation potential was assessed by the expression of DSPP via RT‒PCR. DSPP is considered a significant marker of odontoblastic differentiation. The expression of DSPP in ProRoot MTA was 8.71 ± 1.21 on day 7, and this expression decreased to 3.28 ± 0.35 after 14 days. The expression of DSPP in the Bio MTA + group was 5.38 ± 1.84 on day 7, and this expression significantly increased to 16.85 ± 1.08 after 14 days. A comparison of ProRoot MTA and Bio MTA + revealed that the highest expression of DSPP occurred in the Bio MTA + group after 14 days (16.85 ± 2.06). DMP1, which is an acidic extracellular matrix phosphoprotein, is thought to be involved in matrix mineralization. The mean value of DMP1 gene expression assessed by RT‒PCR in ProRoot MTA was 3.26 ± 1.1 on day 7, and this expression decreased to 1.31 ± 1.03 after 14 days. The expression of DMP1 in the Bio MTA + group (4.08 ± 1.11) on day 7 significantly increased to 9.38 ± 2.03 after 14 days. A comparison of ProRoot MTA and Bio MTA + revealed that the highest expression of DSPP occurred in the Bio MTA + group after 14 days (9.38 ± 2.03). In the early stage of odontoblast differentiation, RUNX2 predominantly regulates the expression of several matrix proteins^21^. The results of RT‒PCR revealed significantly greater expression of RUNX2 on day 7 (*p* < 0.05) in the treated groups of both materials. RUNX2 expression decreases in the late stage of odontogenesis. This was also verified by the results from day 14 in the treated groups. The relative gene expression was normalized to that of GAPDH on days 7 and 14 (Table 3**&** Fig. 9).

**Table 3:** Gene expression in hDPSCs cultured in odontogenic media supplemented with either ProRoot MTA or Bio MTA + on days 7 & 14.

	Day7		Day14		*P* value
	Mean ct	Mean Fold change	Mean ct	Mean Fold change	
RUNX2					
ProRoot MTA	29.52	12.68	25.86	0.34	0.0004
BioMTA+	29.94	12.25	21.36	1.12	0.0001
DSPP					
ProRoot MTA	25.89	8.71	22.67	3.28	0.0001
BioMTA+	28.78	5.38	22.94	16.85	0.0001
DMP1					
ProRoot MTA	30.51	3.26	27.67	1.31	0.003
BioMTA+	31.08	4.08	26.06	9.38	0.004

## Discussion

Pulp capping is a conservative treatment approach in which vital pulp interacts with bioactive materials to preserve the vitality of the pulp tissue while promoting reparative dentin formation^22^. The distinctive characteristics and capacities of dental pulp stem cells (DPSCs) make them crucial in the process of dentin regeneration. Upon the application of biocompatible materials to cover exposed pulp, human dental pulp stem cells (hDPSCs) proliferate, migrate to the site of injury, and eventually undergo odontogenic differentiation with the aim of depositing reparative dentine^17,23^. Optimal pulp-capping material should have the capacity to promote and control the healing process induced by dental pulp stem cells, ogether with adequate physical characteristics, safety to pulp tissue, adequate sealing capability, a rapid setting time, and favorable handling attributes^18^.

Calcium silicate-based materials are widely used in endodontics because of their excellent sealing properties, biocompatibility, and ability to support tissue regeneration^24^. Many studies have extensively documented their properties and potential bioactivity^9,18,24–30^. Nevertheless, the continuous release of novel materials with diverse physical properties on the market necessitates a revised biological and chemical analysis of each compound prior to its clinical application. Thus, in the present study, we aimed to investigate the effects of a rather newly induced calcium silicate-based material, Bio MTA+, in comparison with the widely used ProRoot MTA in terms of viability, proliferation, stemness, and differentiation responses of human dental pulp stem cells to both materials. ProRoot MTA is often used as a benchmark in studies comparing new bioactive materials. This is primarily because its composition is well documented, including its primary components such as tricalcium silicate, dicalcium silicate, tricalcium aluminate, and bismuth oxide. This standardization allows for consistent comparisons with other materials. Moreover, its properties, biocompatibility, and clinical efficacy have been extensively investigated in several studies, which provides a solid basis for comparison^31–35^.

Human dental pulp stem cells (hDPSCs) were employed as a model to replicate clinical settings in which dental pulp must induce differentiation processes to perform dentin repair in the presence of the specified biomaterials. In general, dental stems from different sources exhibit distinctive mesenchymal phenotypes. However, each cell type has distinct responses to different stimuli. For example, multipotent stem cells derived from human exfoliated deciduous teeth, as well as human periodontal ligament stem cells, demonstrate greater osteogenic potential than do hDPSCs. This diversity in properties demonstrates the necessity of conducting a distinct evaluation and classification of the biological behavior of various dental stem cell variants.

In the present study, hDPSCs fulfilled the minimum criteria for the identification of MSCs developed by the International Cellular Therapy Association^36^. The isolated cells were spindle-shaped fibroblast-like cells attached to culture plates, expressed the MSC markers CD90 and CD105, were negative for the hematopoietic marker CD34 and were ultimately able to differentiate into odontogenic, adipogenic and chondrogenic lineages.

The materials used in the present investigation were meticulously selected to encompass different types of pulp capping materials that have not been previously fully evaluated or compared systematically. Bio MTA + is a recently developed innovative alternative to conventional MTA materials. It is designed to provide enhanced manipulation/handling and bioactivity, which makes it a flexible option for a range of endodontic treatments. To the best of our knowledge, our study is one of very few to investigate the biocompatibility and odontogenic differentiation of Bio MTA+. ProRoot MTA is considered a widely used endodontic material known for its superior sealing properties, biocompatibility, and regenerative potential. It promotes the formation of hydroxyapatite, which is essential for the regeneration of dental tissues^26^.

In the present study, both Bio MTA + and ProRoot MTA were prepared as discs according to ISO 10993-5: standards where extracts of the material are prepared and then applied to cultured cells to evaluate their cytotoxicity through diffusion. This preparation is in accordance with similar previous in vitro studies investigating the biological interactions of pulp capping material and dental pulp stem cells and provides insight into the cellular responses to the long-term impact of the tested materials^37–39^.

The impact of cytotoxic compounds derived from pulp-capping materials on cellular viability is highly important. The number of cells that survive is a determining factor in the preservation of pulp vitality following exposure to the materials being utilized. The pulp-capping compounds must either have no biological effect or enhance cellular survival and proliferation. We used a WST-1/CCk-8 assay to evaluate the cytotoxicity of the materials. This assay quantifies the number of viable hDPSCs according to their mitochondrial activity. The increase in absorbance over time can be interpreted as an indirect measurement of cell proliferation, as the metabolic activity of the cells in culture was monitored over time. Our results revealed that the presence of the Bio MTA + extracts resulted in significantly elevated values of absorbance in the CCK-7 assay. hDPSCs exposed to Bio MTA + presented the highest proliferation rate from days 1 to 7, indicating a relatively high level of cytocompatibility. The proliferation of hDPSCs exposed to ProRoot MTA was comparable to that of the control on days 1 and 7 but was significantly greater on day 3. This result was in agreement with that of Esen et al. (2024)^29^. The results of ProRoot MTA testing were consistent with prior research indicating that hDPSCs retain their viability and undergo efficient proliferation when cultured with ProRoot MTA^19,35,38,40^.

We further investigated the impact of both materials on the induction of hDPSCs apoptosis through analysis of Annexin V expression. Annexin V is important for evaluating the cytotoxicity of pulp capping materials because it helps assess the extent of apoptosis in dental pulp cells^41^. Furthermore, the assessment of Annexin V levels serves to guarantee that the selected material induces no apoptosis and thus can improve the long-term efficacy of pulp capping procedures by maintaining the health and functionality of the dental pulp. Notably, the study by Esen et al. investigated only the biocompatibility of Bio MTA + via the WST-1 assay and did not examine the apoptosis and necrotic cell death profiles; this drawback is a limitation of their study. Consistent with the findings from the CCK-8 assay, the results of the Annexin V assay indicated that Bio MTA + caused the least amount of apoptosis, followed by the ProRoot MTA extracts, both of which were superior to the control. Minimal apoptosis levels suggest that the material is biocompatible and promotes the survival of dental pulp cells, which is crucial for effective pulp capping and regeneration^42^. We performed a cell cycle assessment to evaluate the effects of ProRoot MTA and Bio MTA + extraction media on the proliferative activity of hDPSCs. The increase in the percentage of cells in the S and G2/M phases observed with ProRoot MTA and Bio MTA + compared with the control extracts further confirmed that both pulp capping materials enhanced cell differentiation and proliferation. Next, we evaluated the expression of the stemness markers Oct4 and *SOX*2. High expression levels of these markers indicate the maintenance of stemness in hDPSCs, whereas a decrease in their expression suggests a loss of stemness^16,43,44^. Our results revealed that both genes were expressed at higher levels in the Bio MTA + extracts than in the ProRoot MTA extracts, which confirms the regenerative properties of both materials.

Finally, the optimal design of pulp capping materials should aim to augment the odontogenic differentiation capacity of dental pulp stem cells. Thus, we evaluated the expression of markers associated with odontoblastic differentiation (*RUNX2*,* DMP1*,* and DSPP*) via qRT‒PCR. Alizarin Red was used to qualitatively evaluate mineralization. In the early stage of odontoblast differentiation, *RUNX2* predominantly regulates the expression of several matrix proteins. The expression of *RUNX2* in both the BioMTA + & ProRoot groups was greater at the first week and decreased by the end of the second week, which confirms odontogenic differentiation^45^. Dentin sialophosphoprotein (*DSPP*) and dentin matrix protein-1 (*DMP1*) play crucial roles in the effective formation of hard tissues, including teeth and bones. Compared with DMP1, DSPP has a more significant effect on the mineralization of dentin, which controls the organization of the collagen matrix and stimulates the mineralization of the dentin matrix. Our results demonstrated significantly increased expression of both genes in hDPSCs cultured in Bio MTA+, which indicated successful odontogenic differentiation. These findings were consistent with the increase in calcified nodules detected by Alizarin Red staining compared with those in the control group. These results can be attributed to the results of similar studies that described the constant release of calcium and hydroxide ions by calcium silicate-based materials, such as ProRoot MTA. This release in turn leads to the recruitment and proliferation of dental pulp stem cells^46–48^.

The superior performance of Bio MTA + in odontogenic regeneration is closely tied to its unique composition, which includes hydroxyapatite and nanoceramic particles. Hydroxyapatite, a bioactive material that closely resembles the mineral component of natural dentin, enhances the bioactivity of Bio MTA + by promoting mineralization and facilitating cellular attachment, proliferation, and differentiation. This mineral component also contributes to the material’s ability to form a tight seal and induce dentinal bridge formation. Additionally, the inclusion of nanoceramic particles improves the handling properties of Bio MTA+, offering smoother consistency and quicker hydration. These factors collectively contribute to its superior performance in comparison to other pulp-capping materials. For instance, while Biodentine also exhibits high bioactivity and biocompatibility, its faster setting time might limit adaptability in certain clinical scenarios, potentially affecting its interaction with the pulp tissue. In contrast, the slower setting of Bio MTA + allows for more thorough hydration and prolonged release of bioactive ions such as calcium, which are essential for odontogenic differentiation and hydroxyapatite formation^14,46,49^. These results are supported by previous research, confirming the capacity of calcium silicate-based materials to release calcium ions and subsequently promote odontogenic differentiation^37,38,40,50^.

Ultimately, our study faced several limitations. An essential limitation of in vitro experiments, such as those conducted in our study, is their inability to ascertain the precise interaction between the material in question and the host tissue. There is no doubt that in vitro investigations are of utmost importance in the assessment of novel materials. However, consideration should be given when interpreting the findings of these investigations, as the behavior of the investigated materials can be affected by other factors in the clinic. While the in vitro design provided controlled conditions to evaluate Bio MTA+, the lack of interaction with dentin represents a limitation, as dentin plays a critical role in buffering pH, modulating ion exchange, and influencing the biological response of the pulp tissue. Additionally, the study does not account for environmental factors such as pH fluctuations, inflammatory mediators, or variability in clinical settings, all of which could affect the performance and outcomes of Bio MTA + in vivo. Addressing these aspects in future studies would provide a more comprehensive understanding of the material’s clinical applicability and performance. Another limitation we faced arises from the lack of sufficient previous studies that assessed the biological activity and compatibility of Bio MTA+. Further studies of Bio MTA + should be conducted, preferably with an in vivo model to fully appraise the material.

In summary, the results acquired for Bio MTA + confirmed the exceptional level of cytocompatibility, which is comparable to that of ProRoot MTA. This novel direct pulp-capping material demonstrated advantageous bioactivities, such as the preservation of stemness and an increase in the proliferation capacity of hDPSCs. Furthermore, Bio MTA + exhibited favorable bioactive properties by inducing odontogenic differentiation, making it highly promising for use in vital pulp therapy. To build on these findings, future research should focus on conducting long-term in vivo studies to evaluate the material’s performance in real-world clinical scenarios, including its capacity for sustained bioactivity, durability, and impact on pulp vitality over time. Additionally, testing Bio MTA + in dynamic environments that simulate pulpal conditions, such as models that incorporate dentin buffering, inflammatory mediators, and fluctuating pH levels, would provide a more comprehensive understanding of its clinical behavior. These approaches will help bridge the gap between in vitro findings and clinical applicability, further validating Bio MTA + as a reliable choice for regenerative endodontic procedures.

## Data Availability

The datasets used and/or analyzed during the current study are available from the corresponding author on reasonable request.
